# Effort Not Speed Characterizes Comprehension of Spoken Sentences by Older Adults with Mild Hearing Impairment

**DOI:** 10.3389/fnagi.2016.00329

**Published:** 2017-01-10

**Authors:** Nicole D. Ayasse, Amanda Lash, Arthur Wingfield

**Affiliations:** Volen National Center for Complex Systems, Brandeis UniversityWaltham, MA, USA

**Keywords:** speech comprehension, aging, hearing loss, cognitive effort, eye tracking, pupillometry

## Abstract

In spite of the rapidity of everyday speech, older adults tend to keep up relatively well in day-to-day listening. In laboratory settings older adults do not respond as quickly as younger adults in off-line tests of sentence comprehension, but the question is whether comprehension itself is actually slower. Two unique features of the human eye were used to address this question. First, we tracked eye-movements as 20 young adults and 20 healthy older adults listened to sentences that referred to one of four objects pictured on a computer screen. Although the older adults took longer to indicate the referenced object with a cursor-pointing response, their gaze moved to the correct object as rapidly as that of the younger adults. Second, we concurrently measured dilation of the pupil of the eye as a physiological index of effort. This measure revealed that although poorer hearing acuity did not slow processing, success came at the cost of greater processing effort.

## Introduction

The early literature on mental performance in adult aging was largely one of cataloging age-related deficits—most notably, ineffective learning and poor memory retrieval for recent events. It is the case that aging brings changes to the neural structures and network dynamics that carry cognition (Burke and Barnes, 2006; Raz and Kennedy, 2009), with behavioral consequences that include reduced working memory capacity and a general slowing in a number of perceptual and cognitive operations (Salthouse, 1994, 1996; McCabe et al., 2010). This deficit view of aging raises an intriguing paradox when applied to the everyday comprehension of spoken language. This paradox arises from the fact that natural speech runs past the ear at rates that average between 140–180 words per minute (Miller et al., 1984; Stine et al., 1990), that correct word recognition requires matching this rapidly changing acoustic pattern against some 100,000 words in one’s mental lexicon (Oldfield, 1966; see also Brysbaert et al., 2016), and that one must maintain a running memory of the input to connect what is being heard with what has just been heard, and to integrate that with what is about to be heard (van Dijk and Kintsch, 1983).

Given the well-documented cognitive changes that accompany adult aging, surely, understanding spoken language should be among the hardest hit of human skills. Yet, barring significant neuropathology or serious hearing impairment, comprehension of spoken language remains one of the best-preserved of our cognitive functions (Wingfield and Stine-Morrow, 2000; Peelle and Wingfield, 2016). Underlying this success, however, one may still ask: (1) whether such comprehension occurs as rapidly for older adults relative to younger adults; and (2) whether older adults’ success at speech comprehension requires more effort compared to younger adults. These two questions have not heretofore been easy to answer.

A common approach to addressing the first of these questions has been to measure the relative speed with which younger and older adults can indicate the answer to a comprehension or semantic plausibility question after a sentence has been heard. These studies have typically employed a verbal or manual response, such as a key press, to indicate the moment the meaning of the sentence has been understood. Such measures have uniformly implied that older adults are slower in processing speech input than younger adults (e.g., Wingfield et al., 2003; Tun et al., 2010; Yoon et al., 2015). Less clear, however, is the extent to which such off-line, after-the-fact overt responses serve as a true measure of when comprehension has actually occurred (Caplan and Waters, 1999; Steinhauer et al., 2010).

### Eye-Gaze as a Measure of Processing Speed

To address this question, we took advantage of the finding that an individual’s eye-gaze to a picture of an object on a computer screen can be closely time-locked to its reference in a spoken sentence, such that eye-tracking can serve as a useful technique for studying real-time (in-the-moment) speech comprehension (Cooper, 1974; Tanenhaus et al., 2000; Huettig et al., 2011; Wendt et al., 2015; Huettig and Janse, 2016).

Since our question pertains to age differences, it is also fortunate that there are only minimal age differences in the velocity of saccadic eye movements (Pratt et al., 2006). We thus reasoned that measuring both overt responses and eye-gaze responses would allow us to determine whether the assumption that age-related slowing extends to speech comprehension is necessarily correct, or whether estimates of age differences in speed of comprehension have been exaggerated by slowing in the response measures themselves.

Our research strategy was to present younger and older adults recorded sentences that referred to a particular object, with their task being to select, as quickly as possible, the correct one of four pictured objects displayed on a computer screen. Our contrast would be the potential age difference in the time to indicate the referenced object with an overt, off-line selection response, vs. the moment the participants’ eyes fixated on the referenced object as an on-line measure of when the referenced object was actually understood.

In the original “visual world” eye-tracking paradigm participants viewed objects on a computer screen with instructions such as “put the apple that is on the towel in the box”. Using an eye-tracking apparatus that recorded where the eye was fixated on the computer screen, it was found that the participants’ eye gaze moved from object to object as the sentence was being understood as it unfolded in real time (Tanenhaus et al., 1995; see also Cooper, 1974). Subsequent research has recorded time-locked eye-gaze for participants instructed to look at a target picture (e.g., “look at the candle”) to measure the speed of isolating a named target from competitor objects (Ben-David et al., 2011), and tracked eye-gaze when participants have been asked to point to a named object (Hadar et al., 2016) or printed word (Salverda and Tanenhaus, 2010) displayed on a touch screen, or to select a named object by clicking on the correct object picture using a computer mouse (Allopenna et al., 1998). In the present study we used the latter as our overt response measure.

### Pupil Dilation as a Measure of Processing Effort

Pertaining to our second question, a number of behavioral methods have been proposed to measure processing effort. One may, for example, assess the degree of effort by the degree to which conducting a speech task interferes with a concurrent non-speech task (e.g., Naveh-Benjamin et al., 2005; Sarampalis et al., 2009; Tun et al., 2009). Although informative, such dual-task studies are prone to trade-offs in the momentary attention given to each task that may complicate interpretation. Ratings of subjective effort have shown mixed reliability, as well as being an inherently off-line measure (McGarrigle et al., 2014).

To avoid these pitfalls we took advantage of an unusual feature of the pupil of the human eye. Beyond the reflexive change in pupil diameter in response to changes in ambient light, and the discovery that the pupil enlarges with a state of emotional arousal (Kim et al., 2000; Bradley et al., 2008), pupil diameter also increases with control of attention (Unsworth and Robinson, 2016) and increases incrementally with an increase in the difficulty of a perceptual or cognitive task (Kahneman and Beatty, 1966; Beatty, 1982; see the review in Beatty and Lucero-Wagoner, 2000). Importantly, when used while participants are listening to a sentence, pupillometry has the critical advantage of allowing an index of processing effort that does not interfere with performance on the speech task itself (e.g., Kuchinsky et al., 2013; Zekveld and Kramer, 2014).

## Materials and Methods

### Participants

Participants were 20 younger adults (6 men, 14 women) ranging in age from 18 to 26 years (*M* = 21.2 years) and 20 older adults (5 men, 15 women) ranging in age from 65 to 88 years (*M* = 73.6 years). The younger adults were university students and staff and the older participants were healthy community-dwelling volunteers. All participants were self-reported native speakers of American English, with no known history of stroke, Parkinson’s disease, or other neurologic involvement that might compromise their ability to perform the experimental task.

All participants were screened using the Shipley vocabulary test (Zachary, 1986) to insure that any potential age differences in the experimental task would not be due to a chance difference in vocabulary knowledge. As is common for healthy older adults (Kempler and Zelinski, 1994; Verhaeghen, 2003), the older adults in this study had an advantage in terms of vocabulary knowledge relative to the younger adults (*M* older = 16.6, *SD* = 2.43; *M* younger = 13.8, *SD* = 1.71; *t*_(38)_ = 4.01, *p* < 0.001).

Audiometric evaluation was carried out for all participants using a Grason-Stadler AudioStar Pro clinical audiometer (Grason-Stadler, Inc., Madison, WI, USA) by way of standard audiometric techniques in a sound-attenuated testing room. The younger adults had a mean better-ear pure tone threshold average (PTA) of 7.6 dB HL (*SD* = 4.1) averaged across 500, 1000, 2000 and 4000 Hz, and a mean better-ear speech reception threshold (SRT) of 11.4 dB HL (*SD* = 3.9). The older adults had a mean better-ear PTA of 24.7 dB HL (*SD* = 8.7), and a mean better-ear SRT of 25.9 dB HL (*SD* = 8.0). As is typical for their age ranges (Morrell et al., 1996), the older adults as a group had significantly elevated thresholds relative to the younger adults (*t*_(38)_ = 6.14, *p* < 0.001). None of the older adults were regular users of hearing aids.

Vision screening was conducted using a Snellen eye chart (Hetherington, 1954) at 20 feet and the Jaeger close vision eye chart (Holladay, 2004) at 12 inches. All participants had corrected or uncorrected visual acuity at or better than 20/50 for both near and far vision.

This study was carried out in accordance with the approval of the Brandeis University Committee for the Protection of Human Subjects. All subjects gave written informed consent in accordance with the Declaration of Helsinki.

### Stimuli

#### Speech Materials

**Figure 1:**
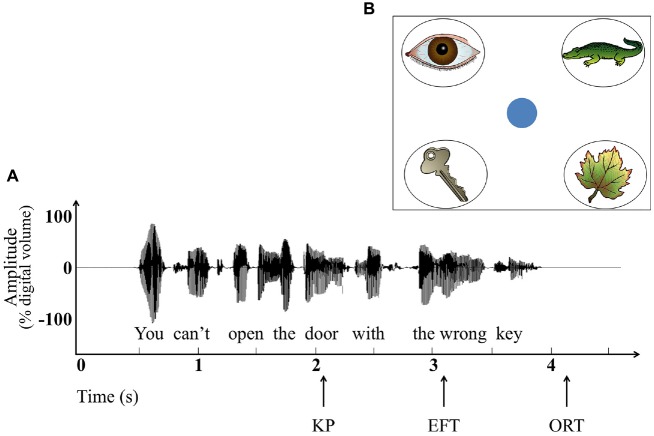
**Experimental stimuli and procedures. (A)** Waveform of an example sentence showing the *knowledge point* (KP) based on a cloze procedure, the relative times after the KP that participants’ eye-fixation indicated knowledge of the target object (*eye fixation time*; *EFT*), and when the target picture was selected with the computer mouse (*overt response time*; *ORT*). **(B)** An example picture array. Depicted in the bottom left corner is the target picture (key), while the other three pictures represent unrelated lures.

The stimuli consisted of 44 sentences recorded by a female speaker of American English. The sentences were spoken with natural prosody and speech rate. The spoken sentences were recorded on computer sound files using Sound Studio v2.2.4 (Macromedia, Inc., San Francisco, CA, USA) that digitized (16-bit) at a sampling rate of 44.1 kHz. Root-mean-square (RMS) amplitude was equated across sentences. Each of the sentences made reference to a picturable object that always formed the last word of the sentence. The waveform of an example sentence is shown in Figure 1A.

Because listeners may continually update their understanding of a sentence as it is being heard, it is possible for the referent of a sentence to be understood before the sentence has been fully completed (Huettig, 2015; Padó et al., 2009). To take this into account, we determined the knowledge point (KP) for each sentence; the point at which a *cloze* procedure conducted in a control study showed that both younger and older adults would know the likely identity of the sentence-final word. As illustrated in Figure 1A, for this example the KP occurred at the word *door*.

The KP was determined for each sentence using a cloze procedure with a separate group of participants (27 younger adults, 9 males and 18 females; *M* age = 20.2, *SD* = 1.20, and 26 older adults, 7 males and 19 females; *M* age = 72.3 years, *SD* = 5.56). Each sentence was presented visually, one word at a time, as participants viewed four object pictures, one of which would be the last word of the sentence being presented. As each word of the sentence was presented participants were asked to indicate if possible which object was being referenced by the sentence. The KP for each sentence was operationally defined as the earliest word in a sentence at which at least 90% of the participants knew the target word. For the majority of sentences, the KP was the same for younger and older adults. (Fifty-five sentences were initially constructed. In 10 sentences the KP differed by one word between the age groups. These sentences were not used in the main experiment, resulting in 44 sentences with age-invariant sentence-final word agreement that would serve as stimuli).

#### Visual Stimuli

For each trial the participants were presented with an array of four pictures of objects displayed in the four corners of a 1280 × 1040-pixel computer screen. Each object was surrounded by a 100-pixel diameter black ring to indicate the area within which the participant would be asked to place the computer cursor to indicate his or her selection. A 50-pixel red fixation circle was centered on the computer screen. Pictures were selected predominantly from the normed color image set of Rossion and Pourtois (2004), supplemented by images taken from clip art databases selected to match the Rossion and Pourtois images in terms of visual style.

In all cases, one of the pictures corresponded to the final word of the sentence that would be heard (*target picture*). The other three pictures (*lure pictures*) were always unrelated to the sentence meaning. None of the lure pictures were phonological competitors for the respective target word, and each set of lure pictures came from distinct functional categories. Figure 1B shows an illustrative stimulus array for the example sentence shown in Figure 1A.

### Procedure

Participants were seated 60 cm from the computer screen with their head placed in a custom-built chin rest to stabilize head movement. Each trial began with the participant positioning the computer cursor on the red fixation circle. This was followed by a 2 s display of the particular four-picture array for that trial to allow the participant to familiarize himself or herself with the pictures and their positions on the computer screen. After the 2 s familiarization period the fixation circle turned blue. This signaled the participant to click on the fixation circle to initiate the sentence presentation. The participant’s instructions were to listen carefully to the sentence and to choose the picture that they believed corresponded to the last word of the sentence as soon as they believed they knew the word. They were to indicate this by using the computer mouse to move the cursor from the fixation circle to the target object and clicking on the mouse to confirm the selection. The computer recorded the moment in time that the participant “clicked” on the correct picture with the mouse (*overt response time, ORT*). Instructions were to respond as rapidly as possible.

Throughout the course of each trial the participant’s moment-to-moment eye-gaze position on the computer screen and changes in pupil size were recorded via an EyeTrac 6000 (Model 6 series, Applied Science Laboratories, Bedford, MA, USA) eye-tracker that was situated below the computer screen and calibrated using EyeTrac software. These data as well as computer mouse movements and response-selection mouse-clicks were recorded via Gaze Tracker software (Eye Response Technologies, Inc., Charlottesville, VA, USA) at a rate of 60 Hz. The sentences and pictures were presented via a custom MATLAB (MathWorks, Natick, MA, USA) program.

The sentences were presented binaurally over Eartone 3A (E-A-R Auditory Systems, Aero Company, Indianapolis, IN, USA) insert earphones. To insure audibility sentences were presented at 25 dB above each individual’s better-ear SRT. The main experiment was preceded by three practice trials using the same procedures as used in the experiment. None of these sentences or pictures was used in the main experiment.

## Results

### Eye Fixations and Overt Response Times

With our procedures we thus had two measures for each sentence presentation: the *ORT*, indicating the participant’s understanding of the sentence by the speed with which they placed the computer cursor and “clicked” on the referenced object on the computer screen, and the *eye fixation time* (EFT): the time point at which the participant’s eye first fixed longer on the correct target picture than on the lures. This latter measure was based on prior studies using eye-tracking (Huettig et al., 2006; Wendt et al., 2014). For each trial, the proportion of time spent fixating on each of the three lures (averaged over the three lures) was subtracted from the proportion of time spent fixating on the target picture in 200 ms time bins (Huettig et al., 2006; Wendt et al., 2014). The EFT was operationalized as the point at which this difference in proportions of fixations exceeded a 15% threshold for 200 ms or more (Wendt et al., 2014, 2015).

The EFTs and the ORTs were measured from the word representing the KP for that sentence. This measure was taken from the midpoint of the KP word to take into account the finding that word recognition often occurs before the full duration of a word has been heard, especially when heard within a sentence context (Grosjean, 1980; Wayland et al., 1989; Lash et al., 2013). Data for incorrect initial target selections were excluded from the analyses (*M* = 6.8% of trials for older adults; *M* = 4.8% of trials for younger adults).

The waveform of the example sentence in Figure 1A shows, along with the KP, the mean EFT on the correct picture, and the mean ORT represented by the mouse-click on the correct object picture. This example is typical in that, for the average participant, the eye fixated on the target picture before the full sentence had been completed, while the overt response occurred shortly after the sentence had ended.

**Figure 2:**
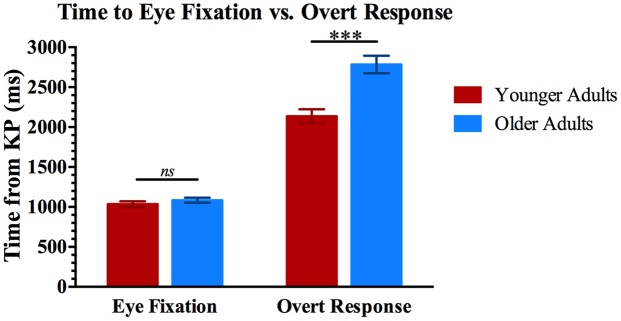
**Results for gaze time and overt responses.** Two vertical bars on the left show mean latencies from the KP in a sentence to the time point when younger and older adults’ eyes fixated longer on the target picture than on the lures (*EFT*). Two vertical bars on the right show the mean latency from the KP in a sentence to the selection of the correct target picture with a computer mouse (*ORT*). Error bars are one standard error. ***Significant pairwise differences, *p* < 0.001.

Figure 2 quantifies these data for the younger and older participants. The results show both an expected finding and a less expected finding based on claims of generalized slowing in adult aging (Cerella, 1994; Salthouse, 1996). The vertical bars on the right side of Figure 2 show the mean latency from the KP in a sentence to the overt response for the younger and older adults. These are exactly the results that would be expected based on generalized slowing in older adults, with the older adults showing significantly longer response latencies than the younger adults (*t*_(38)_ = 4.65, *p* < 0.001). The two vertical bars on the left side of Figure 2 show, for the same participants, the mean latencies from the KP to the time point where listeners’ eye gaze fixated more on the target picture than on the non-target lures. It can be seen that, by this measure, the older adults were no slower in knowing which object was being indicated by the sentence than the younger adults (*t*_(38)_ = 1.01, *p* = 0.32).

This dissociation between knowing the identity of the referenced object, as evidenced by the participant’s eye movements to the target picture, and indicating this knowledge by an overt response, was supported by a 2 (Response type: EFT, ORT) × 2 (Age: Younger, Older) mixed-design analysis of variance (ANOVA), with response type as a within-participants factor and age as a between-participants factor. This confirmed a significant main effect of response type (*F*_(1,38)_ = 447.19, *p* < 0.001, $\eta_{\text{p}}^{2}$ = 0.922), and of age (*F*_(1,38)_ = 18.69, *p* < 0.001, $\eta_{\text{p}}^{2}$ = 0.330), with the dissociation of age effects on the two measures revealed in a significant Response type × Age interaction (*F*_(1,38)_ = 20.51, *p* < 0.001, $\eta_{\text{p}}^{2}$ = 0.351). That is, while older adults may appear slower in comprehending a spoken sentence using a measure that includes decision-making and an overt response (off-line measures that typify reports of age-related slowing in speech comprehension), the eye movement data reveal that the older adults’ time to actually comprehend the semantic direction of a sentence was not significantly slower than younger adults’.

As previously noted, stimuli were presented at a loudness level relative to each individual’s SRT (25 dB above SRT). This procedure was followed to ensure that the stimuli would be audible for all participants. Following the above-cited ANOVA, we conducted an analysis of covariance (ANCOVA) with better-ear PTA as a covariate. This analysis confirmed the same pattern of main effects and the Response type × Age interaction with these effects uninfluenced by hearing acuity. Although confirming that our presentation of the speech stimuli at an equivalent suprathreshold level for each participant was successful in ensuring audibility of the stimuli, this should not necessarily imply that those with better and poorer hearing acuity accomplished their success with equivalent listening effort.

### Pupillometry Measures and Hearing Acuity

To explore the possibility that hearing acuity differences among the older adults may have affected processing effort, we separated the older adult participants into two subgroups based on a median split of hearing acuity.

The *normal hearing* older adult group consisted of the 10 older adults with better hearing acuity, having PTAs ranging from 10 dB HL to 24 dB HL. We use the term “normal” although this group includes individuals with a slight hearing loss (defined as PTAs between 15–25 dB HL; Newby and Popelka, 1992). Although representing thresholds elevated relative to normal-hearing young adults, this range is typically defined in the audiological literature as clinically normal hearing for speech (Katz, 2002).

The *hearing-impaired* older adult group consisted of the 10 older adults with relatively poorer hearing acuity, having PTAs ranging from 26 dB HL to 40 dB HL. These participants’ PTAs lie within the range typically defined as representing a mild hearing loss (26–40 dB HL; see Newby and Popelka, 1992; Katz, 2002).

**Figure 3:**
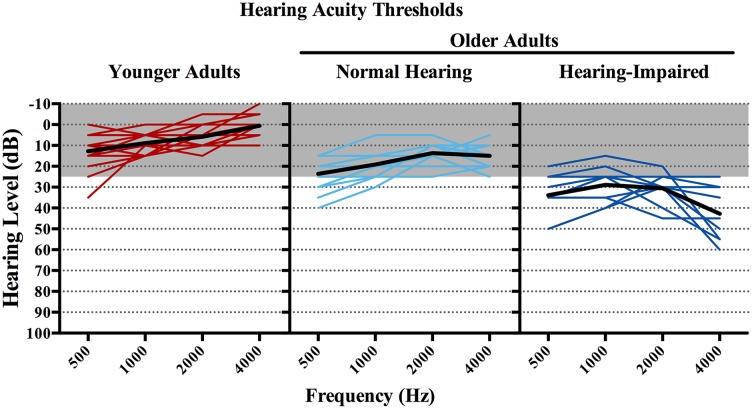
**Better-ear pure-tone thresholds from 500 Hz to 4000 Hz for the three participant group.** Hearing profiles for individual listeners within each participant group are shown in color, with the group average drawn in black. The shaded area in each of the panels indicates thresholds less than 25 dB HL (the range considered clinically normal for speech; Katz, 2002).

The left, middle, and right panels of Figure 3 show better-ear audiometric profiles from 500 Hz to 4000 Hz for the young adults, the 10 normal-hearing older adults and the 10 hearing-impaired older adults, respectively. These data are plotted in the form of audiograms, with the *x-axis* showing the test frequencies and the *y-axis* showing the minimum sound level (dB HL) needed for their detection. Hearing profiles for individual listeners within each participant group are shown in color, with the group average drawn in black. The shaded area in each of the panels indicates thresholds less than 25 dB HL, a region, as indicated above, commonly considered as clinically normal hearing for speech (Katz, 2002).

The normal-hearing and hearing-impaired older adults were similar in age, with the normal-hearing older adults ranging in age from 65 to 88 years (*M* = 73.1 years, *SD* = 7.17) and the hearing-impaired older adults ranged in age from 68 to 81 (*M* = 74.2, *SD* = 4.22; *t*_(18)_ = 0.40, *p* = 0.70). The two groups were also similar in vocabulary knowledge as measured by the Shipley vocabulary test (Zachary, 1986; Normal-hearing *M* = 16.3, *SD* = 2.21; Hearing-impaired *M* = 16.6, *SD* = 2.76; *t*_(18)_ = 0.27, *p* = 0.53).

Pupil size was continuously recorded at a rate of 60 times per second using the previously cited ASL eye tracker (Model 6 series, Applied Science Laboratories, Bedford, MA, USA). routed through the presentation software (GazeTracker, Applied Science Laboratories, Bedford, MA, USA) to allow for pupil size measurements to be synchronized in time with the speech input. Measures of pupil diameter were processed with software written with Matlab 7 (Mathworks, Natick, MA, USA).

Eye blinks were determined by a sudden drop in vertical pupil diameter and were removed from the recorded data prior to data analysis. As is common in pupillometry studies, blinks were defined by a change in the ratio between the vertical and the horizontal pupil diameter. For an essentially circular pupil, the ratio would be approximately 1.0. During a blink or semi-blink the ratio quickly drops toward 0. All samples with a ratio differing more than 1 *SD* from the mean were eliminated (Piquado et al., 2010; see also Zekveld et al., 2010; Kuchinsky et al., 2014; Winn et al., 2015; Wendt et al., 2016).

When comparing relative changes in pupil sizes across age groups it is necessary to adjust for *senile miosis*, where the pupil of the older eye tends to be generally smaller in size, to have a more restricted range of dilation, and to take longer to reach maximum dilation or constriction (Bitsios et al., 1996). To the extent that a change in pupil size is a valid index of processing effort, an absolute measure of a task-evoked pupil size change would thus tend to underestimate older adults’ effort relative to that of younger adults.

To adjust for this potential age difference in the pupillary response, pupil sizes were normalized by measuring, for each individual prior to the experiment, the range of pupil size change as the participant viewed a dark screen (0.05 fL) for 10 s followed by a white screen (30.0 fL) for 10 s. Based on the individual participant’s minimum pupil constriction and maximum pupil dilation, we scaled his or her pupil diameter according to the equation: (*d*_M_ − *d*_min_)/(*d*_max_ − *d*_min_) × 100, where *d*_M_ is the participant’s measured pupil size at any given time point, *d*_min_ is their minimum pupil size (measured during presentation of the white screen), and *d*_max_ is their maximum pupil size (measured during presentation of the black screen; Allard et al., 2010; Piquado et al., 2010). Pupil sizes were additionally adjusted to account for any trial-to-trial variability in pupil diameter (Kuchinsky et al., 2013; Wendt et al., 2016), using a baseline of the mean pupil diameter during a 2-s pre-sentence silence as the *d*_min_ in the above equation and the maximum post-sentence pupil diameter as the *d*_max_.

**Figure 4:**
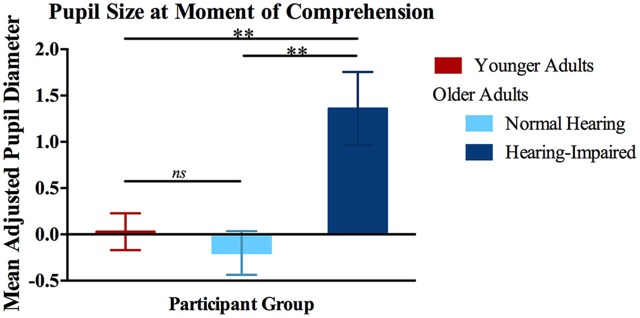
**Mean adjusted pupil diameter leading up to the moment of comprehension.** Pupil diameters calculated over a 1-s window preceding participants’ eye fixations on the target picture. Data are shown for younger adults (left vertical bar), older adults with normal hearing acuity (middle vertical bar), and older adults with hearing impairment (right vertical bar). Error bars are one standard error. **Significant pairwise differences, *p* < 0.01.

Figure 4 shows the accordingly adjusted mean pupil sizes for the three participant groups over a 1-s time window preceding the point of participants’ eye fixation on the correct object picture relative to the lure pictures. This time window was intended to capture the processing effort leading up to this moment (Bitsios et al., 1996).

A one-way ANOVA conducted on the data shown in Figure 4 yielded a significant effect of participant group on pupil diameter (*F*_(2,37)_ = 8.22, *p* = 0.001, $\eta_{\text{p}}^{2}$ = 0.308), with Bonferonni *post hoc* tests confirming that the hearing-impaired older adults showed a significantly greater increase in relative pupil size leading up to their eye fixation on the correct object picture as compared to either the younger adults (*p* = 0.003) or the normal-hearing older adults (*p* = 0.003). The difference in relative pupil sizes between the young adults and the normal-hearing older adults was not significant (*p* = 1.00). This general pattern was seen for pupil sizes at the time of the overt response, although the data were more variable and not statistically reliable.

## Discussion

It has been well documented that older adults are on average slower than their younger adult counterparts on a range of perceptual and cognitive tasks (Cerella, 1994; Salthouse, 1996), to include sentence comprehension when measured by decision latencies indicating that a sentence as been understood (e.g., Wingfield et al., 2003; Tun et al., 2010; Yoon et al., 2015). On the surface our present data would appear to be consistent with an extension of general slowing to spoken language comprehension, at least when comprehension was indexed by latencies to correct response selection. The eye-gaze data, however, tell a different story, one in which on-line comprehension of sentence meaning was accomplished as rapidly for healthy older adults as for younger adults.

### Eye-Gaze as an On-Line Measure

The observed dissociation in this experiment between knowing and the speed of expressing this knowledge in sentence comprehension is consistent with the previously cited distinction suggested by Caplan and Waters (1999). This is the distinction between on-line interpretive processing of a sentence, which may be age-independent for adult listeners, vs. post-interpretive operations, such as planning an action or response, that may well be slower for older adults (see also Waters and Caplan, 2001; Evans et al., 2015). Also implied by this distinction is that an age-independence in on-line interpretive processing may be obscured in sentences that place a heavy demand on working memory for their comprehension. Such working memory demands are associated with sentences that express their meaning with more complex syntax, where older adults are known to show a differential increase in comprehension errors relative to younger adults (e.g., Carpenter et al., 1994; DeCaro et al., 2016) and an increase in the pattern of neural upregulation when comprehension is successful (Wingfield and Grossman, 2006; Peelle et al., 2010).

In this regard, we present our processing-speed data with two caveats. The first is that the sentences used in this study were heard in quiet, were presented at individually adjusted suprathreshold levels, and that they were intentionally, like most of the sentences we hear on a daily basis (e.g., Goldman-Eisler, 1968), grammatically straight-forward and lacking in the working memory demands associated with comprehension of sentences with complex syntax (Just and Carpenter, 1992; Carpenter et al., 1994). As such, these data present a best-case scenario for older adults who, at the perceptual level, have a special difficulty with speech heard in a noisy background (Humes, 1996; Tun and Wingfield, 1999) and who tend to show minimal age difference in comprehension accuracy for grammatically simple sentences (Wingfield and Stine-Morrow, 2000).

Although hearing impairment is known to interact with syntactic complexity when off-line measures of comprehension are employed (e.g., Wingfield et al., 2006), Wendt et al. (2015) have shown that effects of hearing impairment and syntactic complexity can also appear using eye-gaze as an on-line measure. In their experiment, participants heard syntactically simple sentences with a canonical subject-verb-object (SVO) word order, such as, “The little boy greets the nice father” or with the meaning expressed with a less canonical, object-verb-subject (OVS) word order, such as, “It is the nice father that greets the little boy”. As sentences were being heard participants viewed two pictures side-by-side on a computer screen. For this example, one picture depicted a father greeting a little boy and the other depicted a little boy greeting a father. The participant’s task was to indicate with a key press whether the picture on the left or the right of the screen matched the sentence. Wendt et al. (2015) found that eye-fixations to the correct picture tended to be longer for hearing-impaired participants when the relationship between agent and action was expressed with complex syntax.

It may thus be that an absence of age or hearing acuity effects on on-line comprehension speed as demonstrated in the present experiment for syntactically simple sentences might appear when listeners are presented with syntactically complex sentences that place a heavy demand on working memory for their resolution, and perhaps further affected by more challenging listening conditions such as the presence of background noise or especially rapid input rates that are known to place older adults and those with hearing loss at a special disadvantage (see Wingfield and Lash, 2016, for a review of age-related susceptibility to effects of background noise and input rate on speech understanding).

The second caveat is that, in addition to our use of sentences with non-complex syntactic constructions not expected to place significant demands on working memory (see for example Carpenter et al., 1994; DeCaro et al., 2016), the selection of the referenced object on each trial was from a closed set of four possible candidates. Within these constraints, however, the time to older adults’ eye-gaze on the correct object demonstrated that the older adults understood which object was being referenced by the sentence before the sentence had been completed, and that they did so as rapidly as the younger adults.

An alternative to eye-gaze as a measure of on-line sentence processing has been to measure electrical brain activity using event-related potentials (ERPs) as a marker of sentence comprehension. Such studies have primarily focused on the finding that an N400 component of the ERP responds to a semantic violation in a sentence while a P600 component responds to a syntactic violation (see the review in Kutas and Federmeier, 2011). Although many studies have centered on written, as opposed to spoken sentences, and often with such sentences presented in a word-by-word fashion, studies have been conducted that have monitored ERPs as spoken sentences are being heard in real time. One such study in the speech domain revealed affects consistent with our finding of an age-dissociation between on-line vs. off-line measures of sentence comprehension (Steinhauer et al., 2010). These authors found that a P600 was elicited when the syntactic clause boundary in a sentence occurred in one position while the prosodic pattern indicated a different boundary position. They found that the P600 response to this inconstancy occurred as rapidly for older adults as for younger adults, while an off-line measure (responding whether the sentence sounded natural) showed typical age-related slowing. In Caplan and Waters’s (1999) terms, one would characterize this distinction as an age-invariance in on-line interpretive processing vs. the appearance age-related slowing in post-interpretive processing.

### Pupillometry as a Measure of Processing Effort

As we saw, steps were taken to insure that the speech materials were presented at an audible sound level for all participants, such that differences in hearing acuity did not affect either the EFTs to the correct object pictures or the ORTs. This should not imply, however, that this success was achieved with equivalent effort for those older adults with normal hearing or impaired hearing acuity. Indeed, using the pupillary response as a physiological index of processing effort (Piquado et al., 2010; Kuchinsky et al., 2013; Zekveld and Kramer, 2014), however, we found that the older adults with hearing impairment achieved their success at the cost of greater processing effort than required either by the young adults or the older adults with normal hearing acuity.

The underlying connection between effortful processing and the task-evoked pupillary response (TEPR) remains a topic of active investigation. Current evidence suggests that task-related increases in pupil diameter are associated with activity of the *locus coeruleus*-norepinephrine (LC-NE) system, with the LC-NE system serving to modulate prefrontal attentional control (Unsworth and Robinson, 2016). Although pupil dilation is correlated with attention-relation neuronal firing in brain stem *locus coeruleus*, the specific chain of neural events underlying this correlation is complex and not yet fully understood (see “Discussion” Section in Kuchinsky et al., 2014).

At the behavioral level, however, increases in pupil size relative to baseline have been shown to serve as a reliable index of effortful processing, whether in response to listening effort attendant to a degraded speech signal (Zekveld et al., 2011; Kuchinsky et al., 2013; Zekveld and Kramer, 2014; Wendt et al., 2016), to increasing cognitive load in problem-solving and memory tasks (Hess and Polt, 1964; Kahneman and Beatty, 1966; Beatty, 1982) or recall of sentences that increase in length and syntactic complexity (Piquado et al., 2010).

The present study revealed larger adjusted pupil sizes in older adults with impaired hearing, relative to those with normal hearing acuity, in the time period just prior to the point where eye-fixations indicated knowledge of the object being referred to in the sentence. We take these data to support the likelihood that the hearing-impaired participants’ successful comprehension was accomplished with greater effort than the equivalent success of the older adults with better hearing acuity.

This latter finding is especially important in the face of mounting evidence that successful perception of degraded speech can come at the cost of resources that would otherwise be available for encoding what has been heard in memory (Rabbitt, 1968, 1991; Murphy et al., 2000; Wingfield et al., 2005; Surprenant, 2007; Miller and Wingfield, 2010; Cousins et al., 2014) or for comprehension of sentences with complex syntax (Wingfield et al., 2006; DeCaro et al., 2016). This phenomenon represents a “hidden effect” of even a relatively mild hearing loss on older (and younger) adults’ comprehension and recall of spoken input that goes beyond simply missing or mishearing occasional words (Piquado et al., 2012).

## Conclusion

Taken together, our results show that although general slowing may be a hallmark of adult aging, its effects do not apply uniformly across all linguistic operations. Specifically, we found that eye fixations on a referenced object in a spoken sentence occurred as rapidly for older adults as for younger adults, although the older adults were slower in indicating the referenced object with an overt response. As we have indicated, this observed dissociation is consistent with Caplan and Waters (1999) distinction between immediate interpretive processing of sentence meaning that is age-independent, and age-sensitive post-interpretive processes that include decision-making and response selection. An additional finding in this study, however, was that even though the hearing-impaired older adults were no slower in on-line understanding of which object was being referenced by a sentence than older adults with better hearing, their success was accompanied by significantly greater processing effort as indexed by pupil dilation.

The prevalence of hearing impairment among older adults has led to an almost exponential increase in studies of listening effort; how it can be defined and measured (McGarrigle et al., 2014), the cascading effects of front-end perceptual effort on downstream cognitive operations include encoding what has been heard in memory (Wingfield et al., 2005), and a special appreciation for the role modern hearing aids can play in reducing listening effort beyond the historical focus on word recognition *per se* (Sarampalis et al., 2009). This growth of interest in the nature and cognitive costs of effortful listening is well represented in a recent collection edited by Pichora-Fuller et al. (2016).

We have cited studies showing that listening effort attendant to mild hearing loss can affect speech comprehension and effectiveness of encoding what has been heard in memory. Although many older adults may be unaware of this “hidden effect” of hearing impairment on comprehension and immediate memory, there is one consequence of hearing loss that many older adults do recognize. That is, even with a relatively mild hearing loss, many older adults report a sense of stress and end-of-the-day fatigue consequent to the continual effort needed to understanding daily conversational speech (Pichora-Fuller, 2006; Fellinger et al., 2007). This can, in turn, lead to avoidance of social interactions and reduced self-efficacy (Kramer et al., 2002).

In this latter regard, we emphasize the importance of maintaining task engagement by the older adult with or without hearing impairment, even at the cost of cognitive effort. The alternative would be to avoid all difficult tasks that would lead to a potential downward spiral to a general sense of lowered expectations and reduced self-efficacy. We suggest that this was not the case with the hearing-impaired older adults in our study.

An early finding in studies of digit- and word-list recall was that the progressive increase in pupil size as the size of a to-be-recalled list was increased may cease, or reverse, at the point where a list becomes so long as to lead to a memory overload (Kahneman and Beatty, 1966; Peavler, 1974; Granholm et al., 1996). Such an effect can reasonably be interpreted as reflecting task disengagement by the participant when cognitive ability, or one’s willingness to commit effort, is not up to task demands (Kuchinsky et al., 2014; Zekveld and Kramer, 2014). The larger pupil sizes relative to baseline observed for our older hearing-impaired listeners, relative to either the young adults or the better-hearing older adults, suggests that the hearing-impaired older adults in this study remained fully engaged in the experimental task.

### Implications for Interventions

Although our focus is on aging, and age-related hearing loss, it should be noted that mental fatigue due to the continual effort required to successfully understand others’ speech, and its potential effects on cognitive effectiveness, is no less a concern for young adults with hearing impairment (Hicks and Tharpe, 2002), many of whom report being unaware of their hearing loss (e.g., Le Prell et al., 2011).

The frequency-selective amplification and signal processing algorithms available in modern hearing aids can not only improve speech intelligibility, they can also reduce the resource drain associated with effortful listening (Sarampalis et al., 2009). Yet it is the case that two out of three older adults (age 65 and older), and 9 out of 10 younger adults with hearing loss do not use hearing aids, with the numbers especially large in the mild-to-moderate hearing loss range (National Academy on an Aging Society, 1999). Indeed, on average, 10 years pass from the time a person suspects they have a hearing impairment and the time they seek hearing healthcare (Davis et al., 2007).

Numerous studies have been conducted to discover why this most straightforward of interventions has such a low adoption rate. Although cost is certainly a factor, adoption rates remain low even in those countries where hearing aids are available at no cost (Hougaard and Ruf, 2011; Godinho, 2016). This signifies that there are other obstacles beyond cost that must be overcome if we are to increase adoption rates. In some cases, older adults see hearing loss as a natural part of aging and only seek hearing healthcare when the loss becomes severe (van den Brink et al., 1996). Studies have also stressed a stigma associated with wearing hearing aids that is not present, for example, for eyeglasses, with “ageism” an apparent part of this picture (see Hellström and Tekle, 1994; Lundberg and Sheehan, 1994; Levy and Myers, 2004; Jennings, 2005; Southall et al., 2010; Wallhagen, 2010).

It should also be acknowledged that there are “higher-level” auditory processing deficits (Humes, 1996) and attentional factors (Tun and Wingfield, 1995; Tun et al., 2002) that can impair listening effectiveness in older age. As such, setting realistic expectations with the aid of a knowledgeable and trusted audiologist is an essential piece of the full adoption picture. All of these factors have undoubtedly contributed to the discrepancy between hearing aid adoption rates and the dramatic pace of improvements in hearing aid technology.

Among the interventions available for age-associated performance declines, addressing the immediate (Wingfield and Lash, 2016) and long-term (Lin, 2011; Peelle and Wingfield, 2016) cognitive consequences of hearing impairment is among the most direct. The delay in seeking hearing healthcare thus remains a critical public health issue. Creativity in public education that highlights the benefits of reduced listening effort for ease of communication can be an important step in this regard.

## Author Contributions

NDA contributed to experimental design, data collection and analysis, data interpretation, and drafting and revisions of this manuscript. AL contributed to the conception of this work and experimental design, as well as revisions of this manuscript. AW contributed to experimental design, data interpretation, and drafting and revisions of this manuscript.

## Conflict of Interest Statement

The authors declare that the research was conducted in the absence of any commercial or financial relationships that could be construed as a potential conflict of interest.
